# Catalytic Activity of New Oxovanadium(IV) Microclusters with 2-Phenylpyridine in Olefin Oligomerization

**DOI:** 10.3390/ma14247670

**Published:** 2021-12-12

**Authors:** Barbara Gawdzik, Joanna Drzeżdżon, Tatsiana Siarhei, Artur Sikorski, Anna Malankowska, Paweł Kowalczyk, Dagmara Jacewicz

**Affiliations:** 1 Institute of Chemistry, Jan Kochanowski University, Uniwersytecka 7, 25-406 Kielce, Poland; 2 Faculty of Chemistry, University of Gdańsk, Wita Stwosza 63, 80-308 Gdańsk, Poland; joanna.drzezdzon@ug.edu.pl (J.D.); tanya.sergey.rama@gmail.com (T.S.); artur.sikorski@ug.edu.pl (A.S.); anna.malankowska@ug.edu.pl (A.M.); dagmara.jacewicz@ug.edu.pl (D.J.); 3 Department of Animal Nutrition, The Kielanowski Institute of Animal Physiology and Nutrition, Polish Academy of Sciences, Instytucka 3, 05-110 Jabłonna, Poland; p.kowalczyk@ifzz.pl

**Keywords:** oxovanadium(IV) complex, 2-phenylpyridine, oligomerization, 2-chloro-2-propen-1-ol, allyl alcohol, 3-buten-2-ol

## Abstract

So far, few microclusters containing vanadium have been described in the literature. In this report, the synthesis protocol for the preparation of oxovanadium (IV) microclusters with 2-phenylpyridine is shown for the first time. Moreover, the crystal structure of these microclusters is also studied through the use of X-rays. The morphology of the prepared crystals is investigated using a field-emission Scanning Electron Microscope (SEM). The new compound, after activation by modified methylaluminoxane as the catalytic system, is investigated regarding the oligomerizations of 3-buten-1-ol, 2-chloro-2-propen-1-ol, allyl alcohol, and 2,3-dibromo-2-propen-1-ol. The products of oligomerization are tested by the TG-FTIR and MALDI-TOF-MS methods. Moreover, the values of catalytic activities for the new oxovanadium(IV) microclusters with 2-phenylpyridine are determined for the 3-buten-1-ol, 2-chloro-2-propen-1-ol, allyl alcohol, and 2,3-dibromo-2-propen-1-ol oligomerizations. Oxovanadium(IV) microclusters with 2-phenylpyridine are shown to be very highly active precatalysts for the oligomerization of allyl alcohol, 2,3-dibromo-2-propen-1-ol, and 3-buten-1-ol. However, in the case of 2-chloro-2-propen-1-ol oligomerization, the new microclusters are seen as highly active precatalysts.

## 1. Introduction

The metathesis of olefin has been known and used in organic synthesis since the middle of the twentieth century [1,2]. The first example described was the polymerization of norbornene carried out at Du Pont during research on extending the scope of applications of Ziegler-type polymerization [3,4,5]. In 1968, Calderon et al. were the first to use the term “olefin metathesis” [6]. The high synthetic potential of this reaction has remained untapped for years. Initially, the following systems were used as catalysts: TiCl_4_/AlEt_3_, MoCl_5_/AlEt_3_, and WCl_6_/AlEt_3_ [7]. Due to their low cost and simple synthesis, they have played an important role in the industrial use of olefin metathesis—e.g., in the Shell Higher Olefin Process (SHOP) [8]. These have allowed for the efficient course of ring-opening metathesis polymerization (ROMP) and cross-metathesis (CM). However, their usefulness has been limited due to the need for high temperatures and strong Lewis acids. There are the following types of metathesis: Cross Metathesis (CM) [9,10], Ring Closing Metathesis (RCM) [11], and Ring Opening Metathesis (ROM) [12]. In addition, a very important place in the industry is occupied by: Ring Opening Metathesis Polymerization and Acyclic Diene Metathesis (ADMET) polymerization. The synthesis of new, stable complex compounds catalyzing the olefin oligomerization and polymerization reaction, as well as proposing their mechanisms, is a challenge for the next generation of scientists. Many catalytic systems, both homogeneous and heterogeneous, can be used to initiate olefin metathesis [13]. These systems are based on transition metals containing incompletely filled 3D, 4D, or 5D orbitals, such as chromium, vanadium, cobalt, rhenium, or titanium. The use of well-defined catalysts is required in order to obtain compounds of a desired, well-defined structure. These catalysts should be sufficiently stable to be characterized by spectroscopic and X-ray structure methods, and should allow for the control and observation of the formed intermediate products [14]. In turn, the functional groups present in the substrate, as well as the oxygen present in the atmosphere or the environment—e.g., water present in the reaction system—may influence the activity of the catalysts. These factors can react with the active center of the metal, resulting in a partial or complete loss of catalytic properties [15]. They can also compete with the double bond in coordination to the metal ion. Therefore, the reactants used in these reactions must not have functional groups for which the catalyst has a higher affinity than the carbon–carbon double bond. Therefore, mainly organic compounds are used as ligands—e.g., pyridine, lactones, proline derivatives, quinoline, 2,2′-bipyridine, pyrazine, or organophosphorus compounds [16,17,18,19,20,21,22].

The aim of this article is twofold. The first is the development of a synthesis protocol for the preparation of a catalytic system containing oxovanadium(IV) microclusters with 2-phenylpyridine. Secondly, it attempts to optimize the obtained catalytic system, and subsequently to use this system in the olefin oligomerization reaction. The novelty of this work is the synthesis and characterization of the new coordination compound—i.e., oxovanadium(IV) microclusters with 2-phenylpyridine—and, for the first time, the catalytic activity of these microclusters have been investigated for the oligomerization of the following olefins: allyl alcohol, 2,3-dibromo-2-propen-1-ol, 3-buten-1-ol, and 2-chloro-2-propen-1-ol.

## 2. Materials and Methods

### 2.1. Materials

The reagents used for the synthesis of oxovanadium(IV) microclusters with 2-phenylpyridine were as follows: vanadyl acetylacetonate (98% purity), oxydiacetic acid (98% purity), and 2-phenylpyridine (98% purity). All reagents were purchased from Sigma-Aldrich (St. Louis, MO, USA).

The olefins 3-buten-2-ol (97% purity), 2-chloro-2-propen-1-ol (90% purity), allyl alcohol (98.5% purity), and 2,3-dibromo-2-propen-1-ol (90% purity) were purchased from Merck, Darmstadt, Germany. The modified methylaluminoxane MMAO-12 (7 wt.%) aluminum in toluene was purchased from Merck.

### 2.2. Syntheses

The synthesis procedure of oxovanadium(IV) microclusters with 2-phenylpyridine was as follows:

Mix vanadyl acetylacetonate VO(acac)_2_ (2.65 g), H_2_oda (oxydiacetic acid) (1.34 g), and 2.86 mL (0.02 mol) of 2-phenylpyridine, then add 50 mL of water. Boil everything until the solution changes color. The mixture should be refluxed at 100 °C for 2 h. After one month, a new compound was obtained in the form of crystals.

The selected olefins, 3-buten-2-ol, 2-chloro-2-propen-1-ol, allyl alcohol, and 2,3-dibromo-2-propen-1-ol (purchased from Merck), were subjected to oligomerization processes at nitrogen atmosphere, atmospheric pressure, and 65 °C. In the first step, solutions containing 3 µmol of the synthesized complex, 1 mL of toluene, and 1 mL of DMSO were prepared in a glass cell. In the next step, 3 mL of modified methylaluminoxane (MMAO-12, 7 wt.% aluminum in toluene—purchased from Merck) was slowly added into the cell. Next, 1 mL of 3-buten-2-ol, 2-chloro-2-propen-1-ol, allyl alcohol, and 2,3-dibromo-2-propen-1-ol, respectively, were added into the mixture of oxovanadium(IV) microclusters with 2-phenylpyridine activated with modified methylaluminoxane. The solution of each olefin was stirred until a gel was formed.

### 2.3. Physicochemical Characteristics

Diffraction data were collected on an Oxford Diffraction Gemini R ULTRA Ruby CCD diffractometer (T = 295(2) K, MoK_α_ (λ = 0.71073 Å) radiation, Table 1) and were reduced using the CrysAlis RED software (ver. 1.171.41.16a) [23]. The structures were refined and solved using the SHELX package (ver. 2017/1) [24]. H-atoms from water molecules were located on a difference Fourier map and refined using restraints (DFIX command) of *d_(_*_O–H)_ = 0.95 Å and U_iso_(H) = 1.5U_eq_(O), whereas H-atoms bound to N–atoms were located on a difference Fourier map and refined freely. H-atoms bound to C–atoms were placed geometrically and refined using a riding model of *d*_(C–H)_ = 0.93Å and U_iso_(H) = 1.2U_eq_(C). All interactions were identified using the PLATON program (ver. 181115) [25].

The ORTEPII [26], PLUTO-78 [27], and Mercury (ver. 2020.2.0) [28] programs were used to prepare the molecular graphics. Full crystallographic details for the title compound have been deposited in the Cambridge Crystallographic Data Center (deposition No. CCDC 2117153) and they may be obtained from the link: http://www.ccdc.cam.ac.uk (date of access 20 September 2021), via e-mail: deposit@ccdc.cam.ac.uk, or through The Director, CCDC, 12 Union Road, Cambridge, CB2 1EZ, UK.

The MALDI-TOF-MS spectra of oligomerization products were recorded using 2,5- Dihydroxybenzoic acid (DHB) as a matrix. The studies were conducted on a Bruker Biflex III spectrometer.

The TG-FTIR thermogravimetric analysis of oligomerization products was conducted on TG209 Thermometer (Netzsch). All samples were investigated in the temperature range 20–1000 °C.

The morphology of the prepared clusters was investigated using a field-emission Scanning Electron Microscope (SEM). For sample preparation, a certain amount of the powder was deposited onto carbon tape and were imaged with JEOL JSM-7610 F operating at 15 kV.

## 3. Results and Discussion

**Figure 1:**
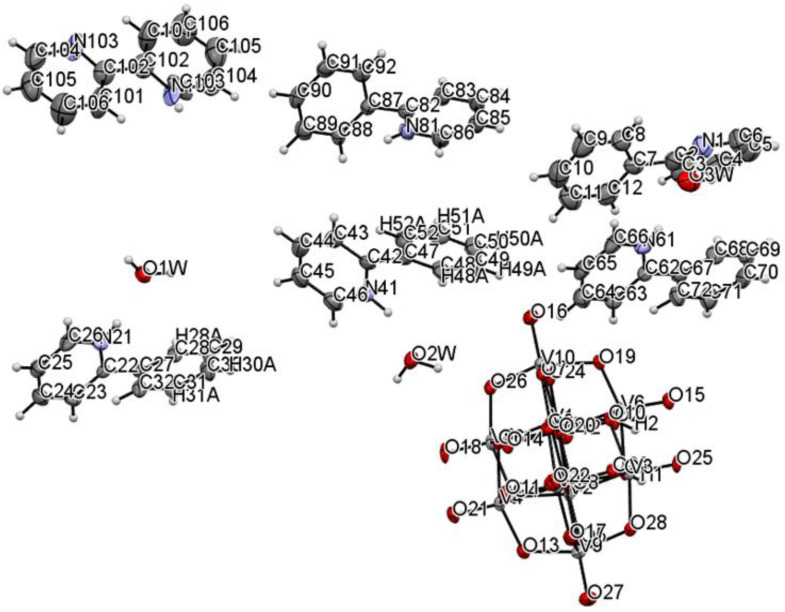
Molecular structure of title compound, showing the atom-labelling scheme.

**Figure 2:**
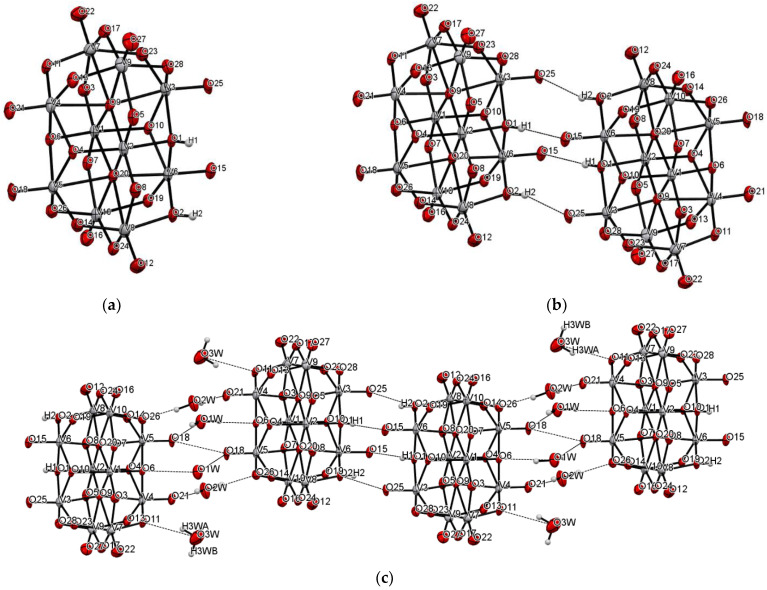

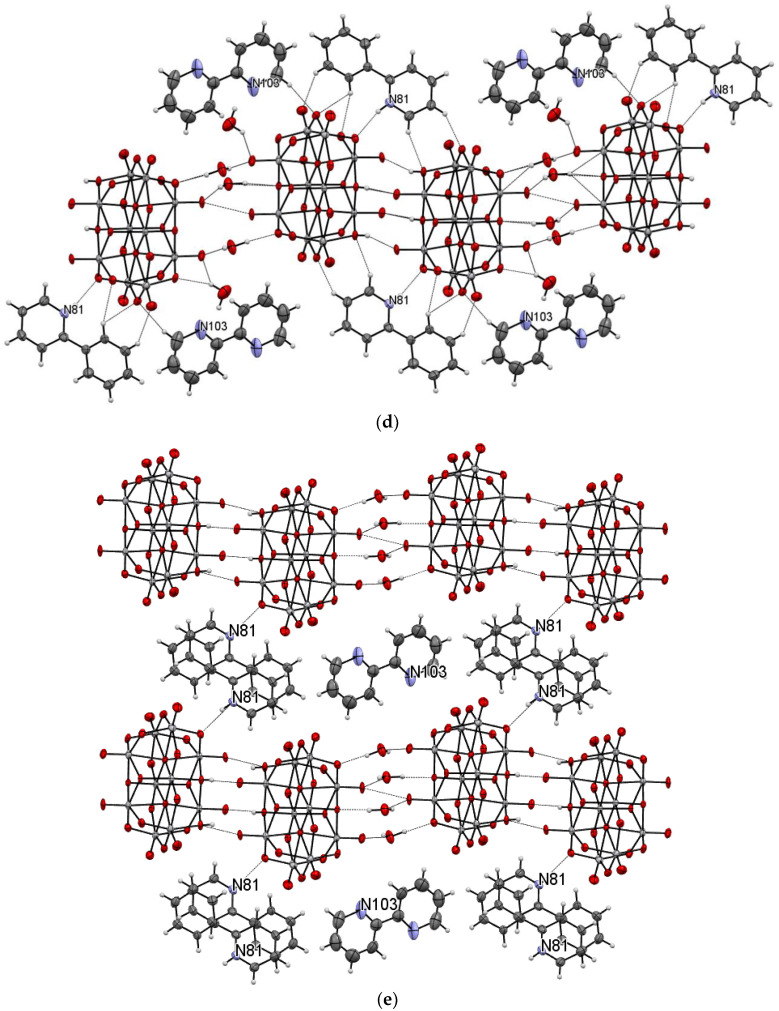
Self-assembly of [V_10_O_28_H_2_]^4−^ cluster (hydrogen bonds are represented by dashed lines); (**a**–**e**) panels exhibit various fragments of new oxovanadium(IV) microclusters with 2-phenylpyridine.

**Figure 3:**
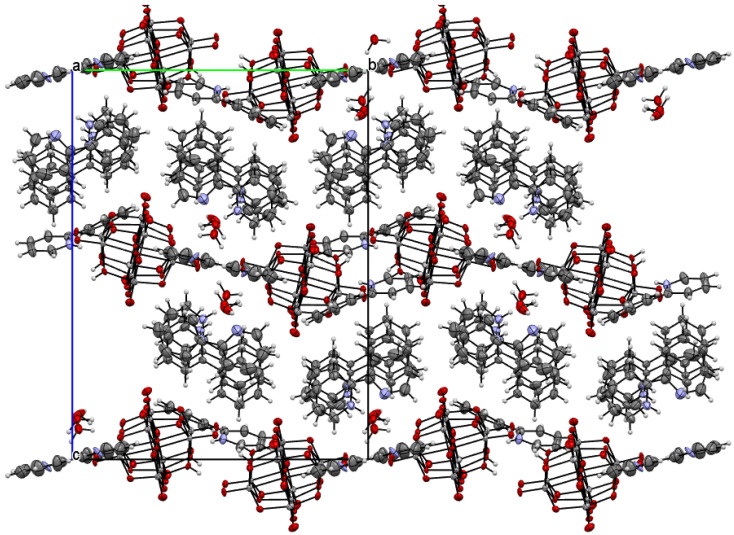
Crystal packing of title compound viewed along the *a*-axis.

The title compound was found to crystallize in the monoclinic *P*2_1_/c space group with one [V_10_O_28_H_2_]^4-^ cluster, four 2-phenylpyridine cations, one and a half 2-phenylpyridine molecules, and three water molecules in the asymmetric unit (Figure 1 and Figure 2, Table 1). In the crystal of the title compound neighboring [V_10_O_28_H_2_]^4-^ clusters interacted through O_(cluster)_–H···O_(cluster)_ hydrogen bonds to form centrosymmetric dimers (Figure 2b, Table 2). The adjacent dimers were connected directly via short O···O contact (*d*_(O18···O18)_ = 2.90 Å) and indirectly through water molecules via O_(water)_–H···O_(cluster)_ hydrogen bonds to produce tapes along *ab-*plane (Figure 2c, Table 2). These tapes are linked by N_(pyridine)_–H···O_(cluster)_, C_(pyridine)_–H···O_(cluster)_ hydrogen bonds, and π-π interactions in which one 2-phenylpiridine molecule and one 2-phenylpyridine cation were engaged to create layers along *a*-axis (Figure 2d,e and Figure 3). The π-stacked columns formed by one 2-phenylpiridine molecule and three 2-phenylpyridine cations were connected with these layers via strong N–H···O_(water)_, O_(water)_–H··· O_(cluster)_, and O_(water)–_H···N_(pyridine)_ hydrogen bonds and weak C_(pyridine)_–H···O_(cluster)_ hydrogen bonds to form a 3D framework structure (Figure 3, Table 2).

**Figure 4:**
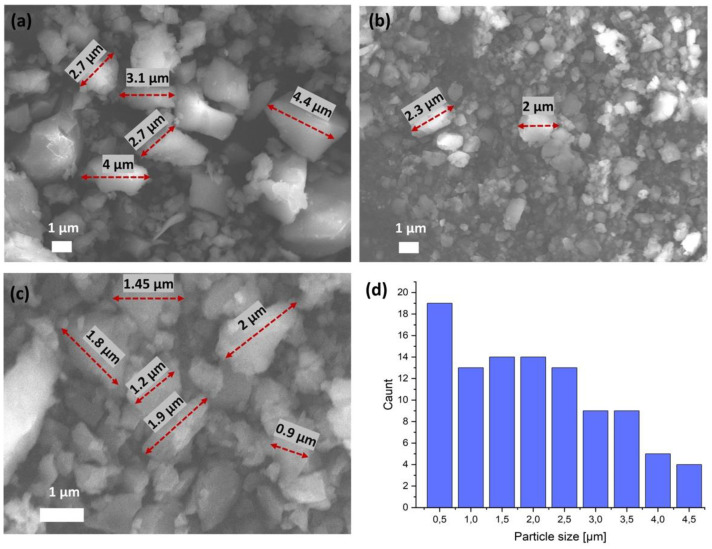
(**a**–**c**) SEM images and (**d**) average size distribution of oxovanadium(IV) microclusters with 2-phenylpyridine.

The field emission SEM images of the particles are shown in Figure 4a–c. SEM is the most widely used technique for characterizing the nanomaterials in terms of physical morphology of the particles. The morphology of the obtained crystals is irregular with a smooth surface. The bigger particle sizes range from 1 to 4.5 µm. It can be noticed that part of the structure has smaller particles, with some sizes of around 0.5 µm. The average size distribution (diameter) of oxovanadium(IV) microclusters with 2-phenylpyridine was calculated from the statistically average sizes of 100 nanoparticles (Figure 4d). No aggregate formation was observed.

**Figure 5:**
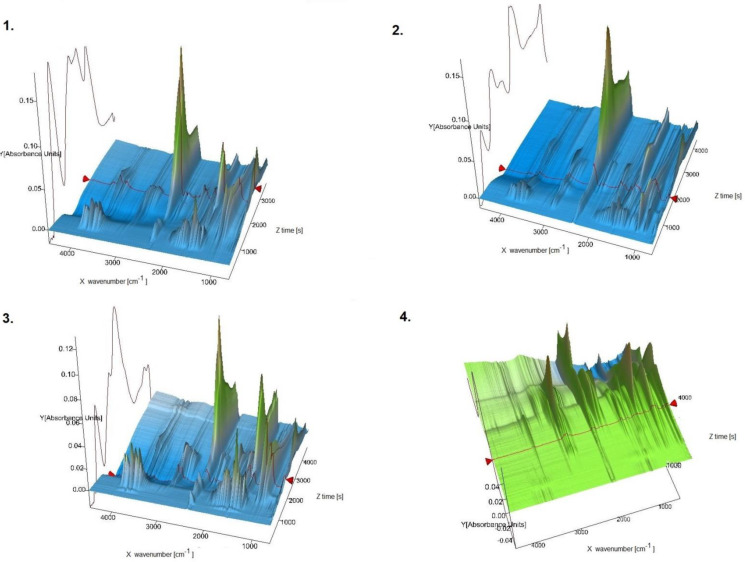
TG-FTIR spectra (3D) for 1—oligomers of 3-buten-1-ol; 2—oligomers of 2-chloro-2-propen-1-ol; 3—oligomers of allyl alcohol; 4—oligomers of 2,3-dibromo-2-propen-1-ol.

In this report, for the first time new oxovanadium(IV) microclusters with 2-phenylpyridine have been used as precatalysts in the oligomerization processes of olefins such as 3-buten-1-ol, 2-chloro-2-propen-1-ol, allyl alcohol, and 2,3-dibromo-2-propen-1-ol. All oligomerization processes were carried out under a nitrogen atmosphere, atmospheric pressure, and temperature of 65 °C. The obtained products from the oligomerization of all the tested olefins, 3-buten-1-ol, 2-chloro-2-propen-1-ol, allyl alcohol, and 2,3-dibromo-2-propen-1-ol, were subjected to TG-FITR and MALDI-TOF-MS tests. The results obtained by the TG-FITR method are shown in Figure 5. For all oligomerization products, water, CO_2_, and CO were released during thermal decomposition. It should be noted, however, that in the case of the oligomers of 2,3-dibromo-2-propen-1-ol, the amounts of water volatilized were insignificant. The oligomerization products of 2,3-dibromo-2-propen-1-ol, during thermal decomposition, after more than 1 h of measurement released HBr. Samples of 3-buten-1-ol oligomers and oligomers of allyl alcohol were characterized by the release of methane after 50 min of measurement. It is worth noting that 2,3-dibromo-2-propen-1-ol oligomers differed in their behavior in the temperature regime from other oligomers. This may be due to the high amount of bromine in the oligomer chain.

The test results obtained by the TG-FTIR method show that the oligomers of 3-buten-1-ol, 2-chloro-2-propen-1-ol, allyl alcohol, and 2,3-dibromo-2-propen-1-ol consist of oligomer chains with a different content of mers; from 3 in the case of 2,3-dibromo- 2-propen-1-ol to 17 mers in the case of allyl alcohol. Oligomeric chains composed of the largest number of mers were obtained using allyl alcohol as the monomer. Thus, a mixture of oligomers consisting of 17, 14, and 11 mers was obtained. The oligomerization of 3-buten-1-ol led to a chain sequence with 16, 14, 12, and 9 mers. In the case of the oligomerization of 2-chloro-2-propen-1-ol, chains of 14, 13, 11, 9, and 7 mers were obtained. A distinctive case was the oligomerization of 2,3-dibromo-2-propen-1-ol, which resulted in the lowest number of mers—i.e., 5, 4, and 3—occurring in the oligomer chains.

These studies on the catalytic activity of new oxovanadium(IV) microclusters with 2-phenylpyridine determined the values of catalytic activity for the compounds formed from the oligomerization of four different olefins: 3-buten-1-ol, 2-chloro-2-propen-1-ol, allyl alcohol, and 2,3-dibromo-2-propen-1-ol. The calculated catalytic activities are as follows in Table 3.

Based on the adopted division of catalytic efficiency introduced in 1999 by Britovsek, Gibson, and Wass [29], the following conclusions can be drawn. The calculated catalytic activities prove that the new microclusters are highly active precatalysts for the oligomerizations of 3-buten-1-ol, allyl alcohol, and 2,3-dibromo-2-propen-1-ol. Moreover, in the case of the oligomerization of 2-chloro-2-propen-1-ol, a new compound of oxovanadium(IV) with 2-phenylpyridine was discovered to be a highly active precatalyst.

## 4. Conclusions

In this report, we described for the first time the synthesis protocol for the preparation of oxovanadium(IV) microclusters with 2-phenylpyridin, and the catalytic activity of the new compound for the oligomerizations of 3-buten-1-ol, 2-chloro-2-propen-1-ol, allyl alcohol, and 2,3-dibromo-2-propen-1-ol. The synthesis of new microclusters was carried out using vanadyl acetylacetonate, oxydiacetic acid, and 2-phenylpyridine in water. The crystallographic studies confirmed that a [V_10_O_28_H_2_]^4-^ cluster forms centrosymmetric dimers by interacting with O_(cluster)_–H···O_(cluster)_. A SEM analysis showed that the morphology of the obtained crystals were irregular with a smooth surface. The bigger particles sizes ranged from 1 to 4.5 µm. The oxovanadium(IV) microclusters with 2-phenylpyridine were used after activation by MMAO-12 as catalytic systems in the oligomerizations of 3-buten-1-ol, 2-chloro-2-propen-1-ol, allyl alcohol, and 2,3-dibromo-2-propen-1-ol. The products of oligomerization were tested by the TG-FTIR and MALDI-TOF-MS methods. TG-FTIR method showed that the oligomer samples obtained from selected olefins release CO, CO_2_, and water. MALDI-TOF-MS data confirmed that all oligomers consist of chains with a different content of mers in the range of 3–17. This study shows the catalytic activities of oxovanadium(IV) microclusters with 2-phenylpyridine act, after activation by MMAO-12, as highly active catalytic systems for 3-buten-1-ol, allyl alcohol, and 2,3-dibromo- 2-propen-1-ol, and that they exhibit highly active catalytic properties for 2-chloro-2-propen-1-ol oligomerization.

## Figures and Tables

**Table 1:** Crystal data and structure refinement for the title compound.

Chemical Formula	V_20_C_121_H_123_N_11_O_62_
Formula weight/g·mol^−1^	3742.10
Crystal system	monoclinic
Space group	*P*2_1_/c
*a*/Å	15.2074(5)
*b*/Å	18.4168(6)
*c*/Å	24.2763(7)
*α*/°	90
*β*/°	91.304(3)
*γ*/°	90
*V*/Å^3^	6797.4(4)
*Z*	2
*T*/K	293(2)
*λ*_Mo_/Å	0.71073
*ρ_cal_*_c_/g·cm^–3^	1.828
*F(000)*	3762
µ/mm^−1^	1.403
*θ* range/°	3.32–25.00
Completeness *θ*/%	99.8
Reflections collected	53,867
Reflections unique	11,941 [R_int_ = 0.1078]
Data/restraints/parameters	11,941/6/1011
Goodness of fit on *F^2^*	1.037
Final R_1_ value (*I* > 2σ(*I*))	0.0639
Final *w*R_2_ value (*I* > 2σ(*I*))	0.0824
Final R_1_ value (all data)	0.1275
Final *w*R_2_ value (all data)	0.0968
CCDC number	2,117,153

**Table 2:** Hydrogen bond geometry for the title compound.

D–H···A	d(D–H) [Å]	d(H···A) [Å]	d(D⋯A) (Å)	∠D–H⋯A (°)
O1–H1···O15 ^i^	0.75(2)	2.03(5)	2.745(5)	175(7)
O1W–H1WA···O6	0.94(2)	2.01(3)	2.908(5)	158(4)
O2–H2···O25 ^i^	0.80(7)	2.05(7)	2.810(5)	159(6)
O1W–H1WB···O18 ^ii^	0.92(4)	2.01(4)	2.910(5)	164(5)
O2W–H2WA···O21 ^iii^	0.93(4)	1.99(5)	2.881(5)	161(5)
O2W–H2WB···O26 ^iv^	0.93(3)	1.88(3)	2.807(5)	172(5)
O3W–H3WA···O11 ^v^	0.95(5)	2.08(6)	3.899(6)	144(4)
O3W–H3WA···O21 ^v^	0.95(5)	2.37(5)	2.160(6)	141(4)
O3W–H3WB···N1	0.93(4)	2.03(5)	2.910(7)	158(6)
N21–H21A···O1W	0.87(5)	1.86(5)	2.718(6)	166(5)
N41–H41A···O2W	0.98(5)	1.77(5)	2.697(6)	156(5)
N61–H61A···O3W	0.76(5)	1.99(5)	2.699(7)	158(5)
N81–H81A···O28	0.80(5)	2.05(5)	2.831(6)	167(5)
C23–H23A··· O23 ^vi^	0.93	2.51	3.334(6)	148
C26–H26A··· O4 ^ii^	0.93	2.20	3.023(6)	147
C28–H28A···O1W	0.93	2.59	3.202(6)	124
C31–H31A···O15 ^vi^	0.93	2.57	3.300(6)	136
C31–H31A···O8 ^iii^	0.93	2.57	3.188(6)	124
C32–H32A··· O10 ^vi^	0.93	2.56	3.480(6)	171
C45–H45A··· O27 ^iii^	0.93	2.57	3.453(8)	158
C46–H46A··· O13 ^iii^	0.93	2.30	2.994(8)	131
C49–H49A··· O16 ^iv^	0.93	2.50	3.300(7)	144
C63–H63A··· O19 ^iv^	0.93	2. 48	3.389(6)	166
C65–H65··· O12 ^i^	0.93	2.55	3.270(7)	135
C66–H66A··· O14 ^i^	0.93	2.56	3.230(6)	129
C68–H68B··· O3W	0.93	2.58	3.384(7)	144
C69–H69A·····O22 ^v^	0.93	2.48	3.353(8)	157
C72–H72A··· O19 ^iv^	0.93	2.57	3.476(7)	166
C83–H83A··· O27 ^vii^	0.93	2.56	3.116(7)	118
C85–H85A··· O24 ^i^	0.93	2.27	3.098(7)	148
C86–H86A··· O2 ^i^	0.93	2.47	3.304(7)	150
C89–H89A···O22	0.93	2.49	2.295(7)	145

Symmetry code: (i) 1-x,1-y,-z; (ii) 1-x,2-y,-z; (iii) x,3/2-y,1/2+z; (iv) 1-x,-1/2+y,1/2-z; (v) x,-1+y,z; (vi) 1-x,1/2+y,1/2-z; (vii) -x,1-y,-z.

**Table 3:** Data for the oligomerizations of selected olefins using new oxovanadium(IV) microclusters with 2-phenylpyridine activated by MMAO-12.

Entry	Olefin	Amt of V (µmol)	t (min)	Molar Ratio Compound: MMAO	Activity (g∙mmol^−1^∙h^−1^)
**1**	3-buten-1-ol	3	35	1:1000	2571
**2**	2-chloro-2-propen-1-ol	3	28	1:1000	643
**3**	allyl alcohol	3	29	1:1000	3724
**4**	2,3-dibromo-2-propen-1-ol	3	25	1:1000	3096

## Data Availability

Full crystallographic details for title compound have been deposited in the Cambridge Crystallographic Data Center (deposition No. CCDC 2117153) and they may be obtained from http://www.ccdc.cam.ac.uk (date of access 20 September 2021), e-mail: deposit@ccdc.cam.ac.uk or The Director, CCDC, 12 Union Road, Cambridge, CB2 1EZ, UK. All other data are stored on the hard drive on the computer and on the portable drive, a backup has been made.

## References

[1] Higman C.S., Lummiss J.A.M., Fogg D.E. (2016). Olefin Metathesis at the Dawn of Implementation in Pharmaceutical and Specialty-Chemicals Manufacturing. Angew. Chem. Int. Ed..

[2] Grubbs R.G., Wenzel A.G., O’Leary D.J., Khosravi E. (2015). Handbook of Metathesis.

[3] Mulhaupt R., Ovenall D.W., Ittel S.D. (1988). Control of composition in ethylene copolymerizations using magnesium mhloride supported Ziegler–Natta catalysts. J. Polym. Sci. Part A Polym. Chem..

[4] Tsujino T., Saegusa T., Furukawa J. (2003). Polymerization of norbornene by modified Ziegler-catalysts. Die Makromol. Chem..

[5] Adisson E., Deffieux A., Fontanille M. (1993). Polymerization of ethylene at high temperature by vanadium-based heterogeneous Ziegler–Natta catalysts. I. study of the deactivation process. J. Polym. Sci. Part A Polym. Chem..

[6] Calderon N., Ofstead E.A., Ward J.P., Judy W.A., Scott K.W. (1968). Olefin metathesis. I. Acyclic vinylenic hydrocarbons. J. Am. Chem. Soc..

[7] Natta G., Dall’Asta G., Mazzanti G. (1964). Stereospecific homopolymerization of cyclopentene. Angew. Chem. Int. Ed..

[8] Mol J.C., Ivin K.J. (1997). Olefin Metathesis and Metathesis Polymerization.

[9] Herbert M.B., Grubbs R.H. (2015). Z-Selective Cross Metathesis with Ruthenium Catalysts: Synthetic Applications and Mechanistic Implications. Angew. Chem. Int. Ed..

[10] Awang N.W., Tsutsumi K., Hustakova B., Yusoff S.F.M., Nomura K., Yamin B.M. (2016). Cross metathesis of methyl oleate (MO) with terminal, internal olefins by ruthenium catalysts: Factors affecting the efficient MO conversion and the selectivity. RSC Adv..

[11] Liniger M., Neuhaus C.M., Altmann K.H. (2020). Ring-Closing Metathesis Approaches towards the Total Synthesis of Rhizoxins. Molecules.

[12] Choinopoulos I. (2019). Grubbs’ and Schrock’s Catalysts, Ring Opening Metathesis Polymerization and Molecular Brushes—Synthesis, Characterization, Properties and Applications. Polymers.

[13] Chołuj A., Nogaś W., Patrzałek M., Krzesiński P., Chmielewski M.J., Kajetanowicz A., Grela K. (2020). Preparation of Ruthenium Olefin Metathesis Catalysts Immobilized on MOF, SBA-15, and 13X for Probing Heterogeneous Boomerang Effect. Catalysts.

[14] Jasiński R., Dresler E. (2020). On the Question of Zwitterionic Intermediates in the [3+2] Cycloaddition Reactions: A Critical Review. Organics.

[15] Schmitt C.C., Belén M., Reolon G., Zimmermann M., Raffelt K., Grunwaldt J.D., Dahmen N. (2018). Synthesis and Regeneration of Nickel-Based Catalysts for Hydrodeoxygenation of Beech Wood Fast Pyrolysis Bio-Oil. Catalysts.

[16] Gawdzik B., Kamizela A., Szyszkowska A. (2015). Lactones with a fragrance properties. Chemist.

[17] Kamizela A., Gawdzik B., Urbaniak M., Lechowicz Ł., Białońska A., Gonciarz W., Chmiela M. (2018). Synthesis, Characterization, Cytotoxicity, and Antibacterial Properties of trans-γ-Halo-δ-lactones. ChemistryOpen.

[18] Drzeżdżon J., Piotrowska-Kirschling A., Malinowski J., Kloska A., Gawdzik B., Chmurzyński L., Jacewicz D. (2019). Antimicrobial, cytotoxic, and antioxidant activities and physicochemical characteristics of chromium (III) complexes with picolinate, dipico-linate, oxalate, 2, 2′-bipyridine, and 4,4′-dimethoxy-2,2′-bipyridine as ligands in aqueous solutions. J. Mol. Liq..

[19] Gawdzik B., Iwanek W. (2005). Synthesis, structure, and stereochemistry of the bora derivatives of 1-[(2-hydroxy-1-naphthyl) methyl] proline. Tetrahedron Asymmetry.

[20] Prezhdo O., Gawdzik B., Zubkova V.V., Prezhdo V.V. (2009). Molecular structure and electrical properties of some phosphonates, phosphine-oxides and phosphates. J. Mol. Struct..

[21] Hearne N., Turnbull M.M., Landee C.P., van der Merwe E.M., Rademeyer M. (2019). Halide-bi-bridged polymers of amide substituted pyridines and -pyrazines: Polymorphism, structures, thermal stability and magnetism. CrystEngComm.

[22] Kwiatek D., Kubicki M., Skokowski P., Gruszczyńska J., Lis S., Hnatejko Z. (2019). Five subsequent new pyridine carboxamides and their complexes with d-electron ions. Synthesis, spectroscopic characterization and magnetic properties. J. Mol. Struct..

[23] Oxford Diffraction Ltd. (2012). CrysAlis CCD and CrysAlis RED.

[24] Sheldrick G.M. (2015). Crystal structure refinement with SHELXL. Acta Crystallogr. C.

[25] Spek A.L. (2009). Structure validation in chemical crystallography. Acta Crystallogr. D.

[26] Johnson C.K. (1976). ORTEP II, Report ORNL-5138.

[27] Motherwell S., Clegg S. (1978). PLUTO-78, Program for Drawing and Molecular Structure.

[28] Macrae C.F., Bruno I.J., Chisholm J.A., Edgington P.R., McCabe P., Pidcock E., Rodriguez-Monge L., Taylor R., van de Streek J., Wood P.A. (2008). Mercury CSD 2.0—New features for the visualization and investigation of crystal structures. J. Appl. Crystallogr..

[29] Britovsek G.J., Gibson V.C., Wass D.F. (1999). The search for new-generation olefin polymerization catalysts: Life beyond metallocenes. Angew. Chem. Int. Ed..

